# Two–Dimensional Disposable Graphene Sensor to Detect Na^+^ Ions

**DOI:** 10.3390/nano11030787

**Published:** 2021-03-19

**Authors:** Hong Gi Oh, Dong Cheol Jeon, Mahmudah Salwa Gianti, Hae Shin Cho, Da Ae Jo, Muhammad Naufal Indriatmoko, Byoung Kuk Jang, Joon Mook Lim, Seungmin Cho, Kwang Soup Song

**Affiliations:** 1 Department of Medical IT Convergence Engineering, Kumoh National Institute of Technology, Gumi 39177, Korea; oh558@naver.com (H.G.O.); vcaptin@kumoh.ac.kr (D.C.J.); algi.salwa@gmail.com (M.S.G.); nunnunnun@naver.com (H.S.C.); jda226@gmail.com (D.A.J.); muhammadnaufal9.1@gmail.com (M.N.I.); 2 Department of Internal Medicine, Keimyung University School of Medicine, Daegu 41931, Korea; jangha106@gmail.com; 3 Department of Creative Convergence Engineering, Hanbat National University, Daejeon 34158, Korea; JoonMookLim@gmail.com; 4 MCK Tech Co., Ltd., Daejeon 34013, Korea; seungmin.cho@mcktech.co.kr

**Keywords:** Na^+^ ion, disposable sensor, fluorinated graphene, reference electrode, fluorobenzene, ISFET

## Abstract

The monitoring of Na^+^ ions distributed in the body has been indirectly calculated by the detection of Na^+^ ions in urine. We fabricated a two–dimensional (2D) Na^+^ ion sensor using a graphene ion–sensitive field–effect transistor (G–ISFET) and used fluorinated graphene as a reference electrode (FG–RE). We integrated G–ISFET and FG on a printed circuit board (PCB) designed in the form of a secure digital (SD) card to fabricate a disposable Na^+^ ion sensor. The sensitivity of the PCB tip to Na^+^ ions was determined to be −55.4 mV/dec. The sensor exhibited good linearity despite the presence of interfering ions in the buffer solution. We expanded the evaluation of the PCB tip to real human patient urine samples. The PCB tip exhibited a sensitivity of −0.36 mV/mM and linearly detected Na^+^ ions in human patient urine without any dilution process. We expect that G–ISFET with FG–RE can be used to realize a disposable Na^+^ ion sensor by serving as an alternative to Ag/AgCl reference electrodes.

## 1. Introduction

Sodium ions (Na^+^) are essential for maintaining the normal functions of the human body, such as transmitting nerve impulses and adjusting the concentration of ions in blood and cells [1,2]. Normally, the concentration of Na^+^ ions in the human body is 135–145 mM [3,4]. However, difficulty in excreting Na^+^ ions due to kidney and gastrointestinal problems or the consumption of Na^+^ ions in excess of the recommended daily intake can lead to hypernatremia (≥145 mM) or hyponatremia (≤135 mM) [5,6]. In general, the concentration of Na^+^ ions in the body is high due to the excessive intake of salt, which causes many diseases, such as confusion, seizures, coma, and adult disease [6]. The 24 h urine analysis method collects urine over 24 h and calculates the concentration of Na^+^ ions distributed throughout the body by determining the concentration of Na^+^ ions in 24 h urine. However, collecting urine over 24 h is very cumbersome. Therefore, there is a need for a disposable sensor capable of detecting Na^+^ ions when urinating without collecting urine over 24 h.

Various methods have been used for the detection of Na^+^ ions, such as atomic absorption spectroscopy (AAS) [7], neutron activation analysis (NAA) [8], flame photometry [9], ion–sensitive optodes (ISO) [10], ion–sensitive electrodes (ISEs) [11], and ion–sensitive field–effect transistors (ISFETs) [12,13,14]. AAS, NAA, and flame photometric methods precisely detect Na^+^ ions. However, these methods require expensive equipment and professional staff for equipment utilization. For ISO, the low cost, high versatility, and compatibility with other assays offer many advantages. However, ISO has a problem that can lead to unwanted cross–responses with changes in the pH of the sample [10]. Conventional ISEs are difficult to miniaturize, and ions tend to leak from the internal solution [15]. These methods cannot be applied to detect Na^+^ ions in disposable sensors when urinating. ISFETs are miniaturized sensors that can quickly detect several different ions and are suitable for use in disposable sensors [11,13,16]. Ag/AgCl reference electrodes (Ag/AgCl–REs) have been widely used in electrochemical ISFETs sensors because the Ag/AgCl electrode exhibits stable potential in electrolytes. However, Ag/AgCl electrodes are conventionally made with fragile glass tubes and require an internal filling solution. For this reason, it is difficult to apply such electrodes to the integrated fabrication process of ISFETs. Hence, a new miniaturized reference electrode that is compatible with the ISFETs manufacturing process is required.

Previously reported Na^+^ ion detection graphene–ISFET (G–ISFET) sensors used Ag/AgCl reference electrodes, and the sensor was a three–dimensional structure (3D) [14,17,18]. In this work, we propose a two–dimensional (2D) sensing structure, a fluorinated graphene reference electrode (FG–RE), for integration with a G–ISFET sensing device for the detection of Na^+^ ions. The 2D structure of G–ISFET integrated with FG–RE was beneficial in detecting Na^+^ ions in human patient urine samples, helping to realize a disposable sensor capable of detecting Na^+^ ions. G–ISFET with FG–RE exhibited linear detection of Na^+^ ions without dilution in human patient urine samples. By integrating FG–RE into the 2D structure of G–ISFET for the detection of target ions, we have developed a new sensing device that addresses the structural limits associated with Ag/AgCl–RE.

## 2. Materials and Methods

### 2.1. Materials and the Fabrication of G–ISFET with ISM

Graphene sheets were purchased from MCK Tech (Daejeon, Korea). Fluorobenzene, sodium ionophore III, polyvinyl chloride (PVC, high molecular weight), bis(1–butylpentyl) adipate, tetrahydrofuran (THF), tris(hydroxymethyl)aminomethane, sodium chloride, potassium chloride, calcium chloride, boric acid, citric acid, trisodium phosphate, Tris base, and hydrochloric acid were purchased from Sigma Aldrich (St. Louis, MO, USA).

The characterization of graphene was performed via Raman spectroscopy (System 1000, Renishaw, Wotton–under–Edge, UK) using an Ar–ion laser at a wavelength of 514 nm. The surface wettability was evaluated based on the water contact angle using the sessile dropping method with a contact angle analyzer (Phoenix 300, SEO Co. Ltd., Suwon, Korea).

The following process was sequentially performed on the pristine graphene sheet transferred onto a polyethylene terephthalate (PET) substrate to fabricate G–ISFET (Supplementary Figure S1). The graphene was washed with ethanol and distilled water to remove residue from the surface. A gold electrode (Au) was deposited to a thickness of 200 nm using a thermal evaporator to form the drain and source electrodes. The length and width of the formed gate channel were 500 and 5000 μm, respectively. To apply bias to the electrodes, conductive wires were bonded to the drain and source electrodes using silver paste. Finally, the drain and source electrodes were covered with epoxy resin to protect the electrodes from the electrolyte. In the case of FG–ISFET, the FG channel was formed by fluorobenzene treatment before wire bonding, followed by wire bonding and shielding with epoxy resin to fabricate FG–ISFET. Briefly, the ion–sensitive membrane (ISM) solution was prepared by dissolving 1 wt.% of sodium ionophore III, 66 wt.% of bis(1–butylpentyl) adipate, and 33 wt.% of PVC in 1 mL of THF [17]. Then, 3 μL of the prepared ISM solution was dropped onto the channel surface of G–ISFET to fabricate the Na^+^ ion sensor (G–ISFET–ISM), which was then stored at 25 °C for 24 h to dry THF.

### 2.2. Detection of Na^+^ Ions Using G–ISFET–ISM

The transfer characteristics of G–ISFET were characterized using two digital sourcemeters (Keithley 2400, Keithley, Cleveland, OH, USA). Either Ag/AgCl–RE or FG–RE was used as a gate electrode for gate bias on G–ISFET in the electrolyte solution. G–ISFET–ISM was submerged in a 100 mM NaCl solution for 30 min to activate the ISM, followed by immersion in DI water for 15 min before Na^+^ ion detection [18]. In order to evaluate the Na^+^ ion sensitivity of G–ISFET–ISM, Tris–HCl buffer (50 mM, pH 7.4) was used. NaCl, KCl, and CaCl_2_ were dissolved in Tris–HCl buffer, and their concentrations were adjusted to the range of 10^−4^–10 M. Carmody buffer (0.2 M boric acid, 0.05 M citric acid, and 0.1 M trisodium phosphate) was used as a pH buffer solution. We evaluated the sensitivity using at least 20 disposable sensors independently in each experiment, and all statistical analysis results are presented as the mean ± standard deviation.

We performed Na^+^ ion detection in real patient urine samples. The real human patient urine samples were provided by Keimyung University Dongsan Hospital (Daegu, Korea) and approved by the Institutional Review Board of Keimyung University Dongsan Hospital (IRB No. 2015-03-018). The information on the 4 real human urine samples is summarized in Table 1. All the real patient urine samples were used without any additional dilution process.

## 3. Results and Discussion

### 3.1. Fluorinated Graphene

Fluorobenzene has a structure in which one hydrogen atom on the benzene ring is replaced with a fluorine atom. Fluorobenzene can thus easily interact with the graphene surface through π–π interactions [19]. Therefore, we performed fluorobenzene treatment for the functionalization of pristine graphene to FG. For fluorine functionalization, graphene was immersed in 99% fluorobenzene for 30 s and then completely dried at 25 °C.

**Figure 1:**
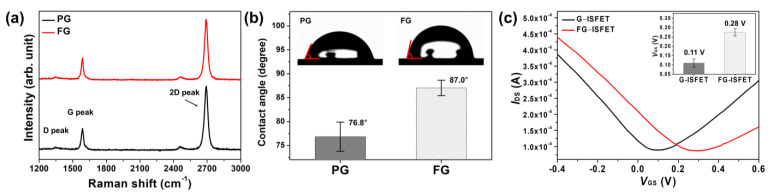
The characteristics of graphene before and after fluorobenzene treatment for 30 s: (**a**) Raman spectra; (**b**) water contact angles; and (**c**) *I*_DS_–*V*_GS_ of G–ISFET and FG–ISFET.

The Raman spectra of graphene are shown in Figure 1a. The D, G, and 2D peaks of pristine graphene (PG) were observed at 1348.0, 1586.1 and 2688.5 cm^−1^, respectively. The intensity ratios, IG/I2D and ID/IG, of PG were 0.34 and 0.14, respectively, which indicates that the graphene sample was a single layer with few defects [20,21]. For FG, the D, G, and 2D peaks were observed at 1349.6, 1587.6 and 2689.8 cm^−1^, respectively. The intensity ratios, IG/I2D and ID/IG of FG, were 0.37 and 0.15, respectively. There were no significant differences between the Raman spectra of FG and PG. Graphene was fluorinated by fluorobenzene without changing the graphene structure, because benzene binds to graphene through π–π interactions rather than ionic bonds. However, for fluorinated graphene with plasma treatment, the G and 2D peaks were shifted owing to the C–F ionic bond after fluorination [22]. The water contact angles of PG and FG were 76.8° and 87.0°, respectively, as shown in Figure 1b. The water contact angle of FG was higher, owing to the fluorine atom of fluorobenzene on FG [23].

### 3.2. Characteristics of G–ISFET and FG–ISFET

Characterization of G–ISFET and FG–ISFET with Ag/AgCl–RE were conducted according to the drain–source current (*I*_DS_), drain–source voltage (*V*_DS_), and gate–source voltage (*V*_GS_) in a Tris–HCl buffer solution. The *V*_DS_ value was fixed at 0.05 V, and *V*_GS_ was swept from −0.6 to 0.6 V with a 2.5 mV step. Figure 1c shows the *I*_DS_–*V*_GS_ of G–ISFET and FG–ISFET. The Dirac point (*V*_Dirac_) of the G–ISFET was 0.11 V. In contrast, for the FG–ISFET, *V*_Dirac_ was 0.28 V. *V*_Dirac_ of G–ISFET shifted to more positive values (by 0.17 V) after fluorobenzene treatment, because *V*_Dirac_ of G–ISFET shifts depending on the functional groups or doping state on the surface [24,25]. Graphene follows an sp^2^ hybridized carbon structure and has a delocalized electron cloud due to the π electrons of each carbon atom [26]. Benzene rings also have a delocalized electron cloud due to π electrons and a relatively low electron density compared with graphene. Graphene and fluorobenzene are bound via π–π interactions by electrostatic forces, and the electron density of graphene is decreased owing to the attraction of π electrons away from graphene [25]. Thus, *V*_Dirac_ of FG–ISFET shifted towards positive values due to the p–doping effect.

An ideal reference electrode should maintain a constant potential over long–term use and should not be sensitive to specific ions in the electrolyte [27]. Graphene is sensitive to pH and cations (Na^+^ and K^+^) due to the hydroxyl groups on its surface [28,29]. FG–ISFET has been reported to not exhibit sensitivity to pH [19]. However, FG–ISFET has not yet been sufficiently investigated for its sensitivity to other ions.

**Figure 2:**
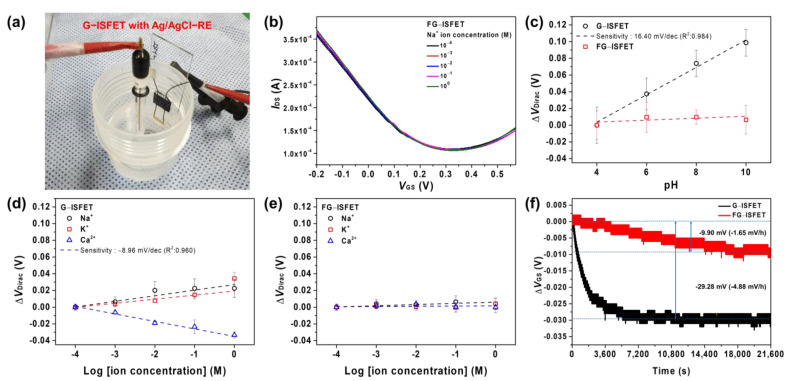
Evaluation of sensitivity of G–ISFET and FG–ISFET with Ag/AgCl–RE. Linear fits were used to extract sensitivity: (**a**) experimental setup using G–ISFET with Ag/AgCl–RE; (**b**) *I*_DS_–*V*_GS_ of FG–ISFET depending on Na^+^ ion concentration; (**c**) pH sensitivity of G–ISFET and FG–ISFET; (**d**) Na^+^, K^+^, and Ca^2+^ sensitivity of G–ISFET; (**e**) Na^+^, K^+^, and Ca^2+^ sensitivity of FG–ISFET; and (**f**) long–term stability of G–ISFET and FG–ISFET in Tris–HCl buffer, in which 100 mM NaCl was dissolved for 6 h.

We evaluated the sensitivity of G–ISFET and FG–ISFET with Ag/AgCl–RE to cations (Na^+^, K^+^, and Ca^2+^ ions) in a Tris–HCl buffer solution, as shown in Figure 2a. The size of slide glass was 76 × 24 × 2.0 mm^3^. The sensitivity of G–ISFET and FG–ISFET to cations in the buffer solution was evaluated by the change in *V*_Dirac_. The typical *I*_DS_–*V*_GS_ plots of FG–ISFET are shown in Figure 2b. FG–ISFET was not sensitive to Na^+^ ions, as evidenced by *V*_Dirac_, which did not shift with changes in the Na^+^ concentration in the buffer solution. Moreover, FG–ISFET was not sensitive to pH, as shown in Figure 2c. However, G–ISFET was sensitive to pH at 16.40 mV/pH (Supplementary Figure S2). For G–ISFET, *V*_Dirac_ became more positive with increasing Na^+^ and K^+^ ion concentrations, as shown in Figure 2d. In the case of Ca^2+^ ions, *V*_GS_ became more negative with the increasing Ca^2+^ ion concentration (−8.96 mV/dec). However, FG–ISFET did not exhibit shifts to Na^+^, K^+^, and Ca^2+^ ions in the buffer solution, as shown in Figure 2e. Fluorobenzene prevented the reaction with ions in the electrolyte by blocking the active groups on the graphene channel surface.

**Figure 3:**
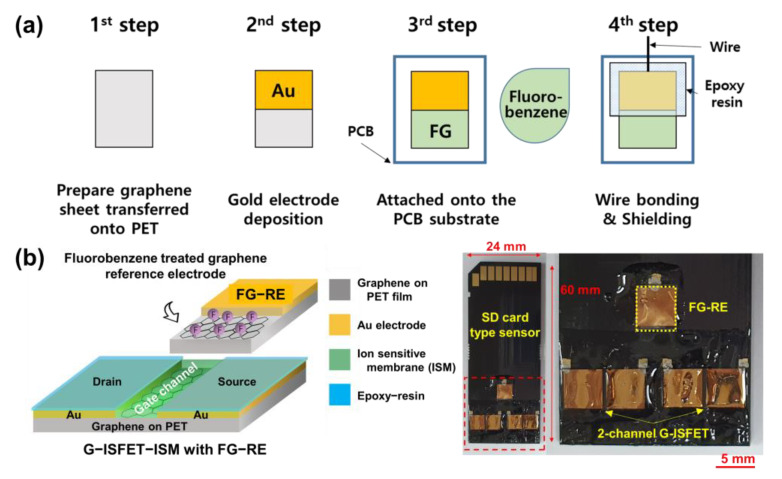
Schematic diagram and image of the 2D structural sensing device: (**a**) fabrication process of the fluorinated graphene reference electrode (FG–RE); (**b**) G–ISFET–ISM and FG–RE were integrated on an SD card–type printed circuit board (PCB).

To evaluate the long–term stability of G–ISFET and FG–ISFET, Δ*V*_GS_ was determined in real time using a Tris–HCl buffer, in which 200 mM NaCl was dissolved for 6 h. The *V*_DS_ value was fixed at 0.05 V, and the *I*_DS_ value was fixed at 180 μA. G–ISFET exhibited a rapid change in Δ*V*_GS_ (−29.28 mV), and such value plateaued after 1.5 h, as shown in Figure 2f. In contrast, Δ*V*_GS_ of FG–ISFET was −9.90 mV, and such value plateaued after 4.4 h. This Δ*V*_GS_ value would be negligible throughout the sensor sensitivity evaluation time (30 s). FG–ISFET was not sensitive to cations (H^+^, Na^+^, K^+^, and Ca^2+^) and was stable for 6 h. Based on these results, we fabricated an FG reference electrode (FG–RE), as shown in Figure 3a. The FG sample was 500 × 5000 μm^2^ in size. To integrate FG–RE and G–ISFET to the 2D structure, a printed circuit board (PCB) of the type that can be inserted into a secure digital (SD) card slot was manufactured, as shown in Figure 3b. Two G–ISFETs and one FG–RE were fabricated on the PCB tip (60 × 24 × 2.1 mm^3^). There was a gate channel between drain and source electrodes of G–ISFET. G–ISFET operated as a solution gate–FET through FG–RE, and FG–RE was 9.5 mm away from G–ISFET. G–ISFET operates as a sensor that detects ions in electrolyte solution, and FG–RE replaces the Ag/AgCl reference electrode and applies a gate bias in the solution to induce the electric field effect of G–ISFET.

### 3.3. G–ISFET with ISM to Detect Na^+^ Ions Using Ag/AgCl–RE or FG–RE

**Figure 4:**
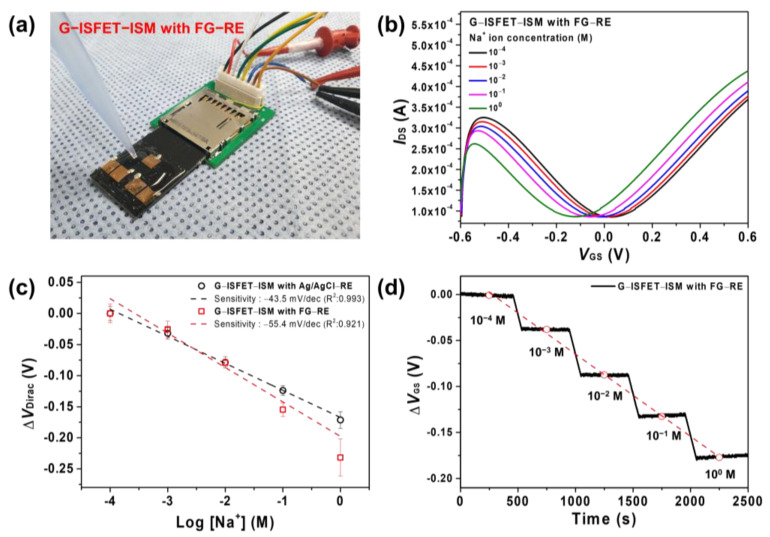
(**a**) Experimental setup using G–ISFET–ISM with FG–RE; (**b**) *I*_DS_–*V*_GS_ of G–ISFET–ISM with FG–RE depending on Na^+^ ion concentration; (**c**) the sensitivity of G–ISFET–ISM with Ag/AgCl–RE or FG–RE to Na^+^ ions. Linear fits were used to extract sensitivity; (**d**) real–time detection of Na^+^ ions using G–ISFET–ISM with FG–RE.

The gate channel of G–ISFET was covered with ISM and was employed to detect Na^+^ ions using Ag/AgCl–RE or FG–RE in Tris–HCl buffer solution. Figure 4a depicts G–ISFET–ISM with FG–RE in use during a typical experimental run. The sensing device had a 2D structure, and the testing solution was dropped onto the 2D sensing device using a micropipette. We showed the *I*_DS_–*V*_DS_ characteristics of G–ISFET–ISM with Ag/AgCl or FG–RE. *V*_DS_ was increased from 0.0 V to 0.05 V in a Tris–HCl buffer solution; *I*_DS_ increased with respect to *V_GS_* at the *n*–channel region (Supplementary Figure S3). When gate voltage was applied by using FG–RE, ions in the electrolyte moved and formed electrical double layers on gate channel surface of G–ISFET–ISM and FG–RE. The electrical double layers had no charge transfer, and current flow through the electrolyte from the G–ISFET–ISM to the FG–RE was negligible (<0.1 nA). This indicates that the electrical double layers act as the thin insulators in both sides. However, when FG–RE was used at the same *V*_GS_ voltage, the current value was smaller when Ag/AgCl–RE was used. The transconductance (*g*_m_) of G–ISFET–ISM was higher when Ag/AgCl–RE (0.3 mS) was used than when FG–RE (0.18 mS) was used.

Clinically, the concentration of Na^+^ ions in urine is 10–250 mM [30]. The *I*_DS_–*V*_GS_ plot of G–ISFET–ISM with FG–RE indicated ambipolar graphene field effect transistor (FET) behavior (*p*–channel and *n*–channel), which is a typical characteristic of G–ISFET with Ag/AgCl–RE. *V*_Dirac_ of G–ISFET–ISM with FG–RE was shifted by −55.4 mV/dec depending on the Na^+^ ion concentration, as shown in Figure 4b. The sensitivity of G–ISFET–ISM with Ag/AgCl–RE was −43.5 mV/dec (Supplementary Figure S4a). G–ISFET–ISM with either Ag/AgCl–RE or FG–RE exhibited a linear sensitivity to Na^+^ ions in the range of 10^−4^–10^0^ M, as shown in Figure 4c. Na^+^ ions were captured on the graphene surface through ISM, and the ion charge on the graphene surface increased as the concentration of Na^+^ ions increased. As the concentration of Na^+^ ions increased, the positive charge on the graphene surface increased; hence, *V*_Dirac_ of G–ISFET–ISM shifted towards negative values.

The voltage between G–ISFET–ISM and FG–RE was set with respect to the ISM gate channel and FG–RE. Considering *V*_GS_ in FG–RE, the change of surface charge in the ISM gate channel resulted in the variation of voltage between the ISM gate channel and FG–RE. The bulk potential of the electrolyte solution was determined by *V*_GS_ in FG–RE with electrostatic equilibrium and capacitive coupling [20]. Therefore, the voltage between the ISM gate channel and FG–RE was the only parameter related to the concentration of Na^+^ ions in the electrolyte solution. The change of Na^+^ ions concentration in the electrolyte solution led to the variation of surface charge by the capture of Na^+^ ions on the ISM gate channel and modulated the channel conductance of ISM gate channel in G–ISFET–ISM. The variation of *V*_Dirac_ on G–ISFET–ISM can be expressed as follows:

Δ*V*_Dirac_ = (*V*_ISM_ − *V*_S_) − (*V*_FNa+_ + *V*_F_ − *V*_S_) (1)

where *V*_ISM_ is the Na^+^ ion sensitivity of the ISM gate channel, *V*s is the potential of the source electrode, *V*_FNa+_ is the Na^+^ ion sensitivity of FG–RE, and *V*_F_ is the bias potential of FG–RE. The Na^+^ ions sensitivity of G–ISFET–ISM is determined by the differential response between the ISM gate channel (*V*_ISM_) and FG–RE (*V*_FNa+_). The *I*_DS_–*V*_DS_ characteristics of G–IGFET–ISM with FG–RE in the Tris–HCl buffer solution, where *V_GS_* was fixed at 0.5 V and *V*_DS_ ranged from 0.0 V to 0.05 V, are shown in the Supplementary Figure S3b. *I*_DS_ was increased depending on the Na^+^ ion concentration in electrolyte solution. This was consistent with the *V*_Dirac_ value shifting to the left as the Na^+^ ion concentration increased, as shown in Figure 4b.

We also conducted real–time detection of Na^+^ ions in Tris–HCl buffer. The results of the real–time detection of Na^+^ ions using G–ISFET–ISM with FG–RE are shown in Figure 4d. For this, ∆*V*_GS_ was continuously measured with fixed *V*_DS_ (0.05 V) and *I*_DS_ (180 μA) values while adjusting the Na^+^ ion concentration by periodically injecting a high–concentration NaCl solution. G–ISFET–ISM with FG–RE exhibited an immediate and linear response in real time to changes in the Na^+^ ion concentration. We showed the previously published studies to fabricate a graphene–based ion sensor in Table 2. Some studies used Ag/AgCl electrode, gate–free, HfO_2_, Ag, and Pt wire as the reference electrodes and accurately detected many kinds of ions. However, we fabricated a 2D sensing structure using FG–RE and G–ISFET–ISM to realize the portable Na^+^ ions sensor in this work. Our 2D sensing structure detected Na^+^ ions in the Tris–HCl buffer solution with high reproducibility.

**Figure 5:**
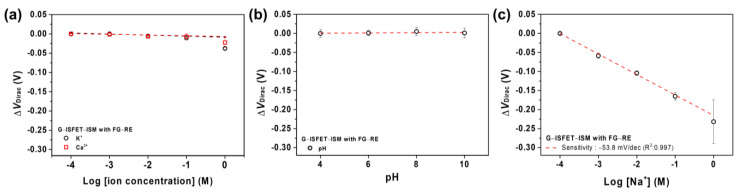
(**a**) Sensitivity of G–ISFET–ISM with FG–RE to K^+^ and Ca^2+^ ions in Tris–HCl buffer, in which 100 mM NaCl was dissolved. Linear fits were used to extract sensitivity; (**b**) sensitivity to pH in Carmody buffer; and (**c**) sensitivity to Na^+^ ions in Tris–HCl buffer, in which 100 mM KCl was dissolved.

Normally, many kinds of ions in the body can be released through urine. The release of such ions depends on food intake and kidney health [30,41]. Therefore, we evaluated G–ISFET–ISM with FG–RE in the presence of interfering ions (H^+^, K^+^, and Ca^2+^). The evaluation of the sensitivity of G–ISFET–ISM with FG–RE to K^+^ and Ca^2+^ ions was performed using a Tris–HCl buffer solution, in which 100 mM NaCl was dissolved. The ∆*V*_Dirac_ values of G–ISFET–ISM were considerably lower for K^+^ and Ca^2+^ ions compared to those for Na^+^ ions, as shown in Figure 5a. Because the ISM selectively allows passage of Na^+^ ions through the membrane according to the selectivity coefficient, the interfering effects of K^+^ and Ca^2+^ greatly decrease in the presence of Na^+^ ions in the electrolyte [13,42]. The results of the evaluation of G–ISFET–ISM with Ag/AgCl–RE with regards to interfering ions are shown in Supplementary Figure S5. Figure 5b shows the sensitivity of G–ISFET–ISM with FG–RE to pH. As shown in Figure 2c, G–ISFET exhibited sensitivity to pH, due to the oxygen functional groups on the graphene channel surface [22,43]. However, G–ISFET–ISM did not exhibit sensitivity to pH. The gate channel surface of G–ISFET–ISM that was exposed to the buffer solution was covered by the ISM; thus, the oxygen functional groups did not react with H^+^.

Figure 5c shows the sensitivity of G–ISFET–ISM with FG–RE to Na^+^ in the Tris–HCl buffer solution, in which 100 mM KCl was dissolved. G–ISFET–ISM with FG–RE exhibited a sensitivity of −53.8 mV/dec despite the high concentration of K^+^ ions in the buffer solution. We characterized the sensitivity of G–ISFET–ISM with FG–RE to glucose, lactate, bicarbonate, and Mg^2+^. G–ISFET–ISM was insensitive to glucose, lactate, bicarbonate, and Mg^2+^ (Supplementary Figure S6). Hence, G–ISFET–ISM with FG–RE exhibited high selectivity for Na^+^ ions.

### 3.4. Urine Test

We evaluated the sensitivity of G–ISFET–ISM with FG–RE in human patient urine samples. The patient urine samples were provided by Keimyung University Dongsan Hospital and were used without any dilution process. Information of the Na^+^, K^+^, Cl^−^, and Cr^3+^ ions concentration in each urine sample is summarized in Table 1. The ion concentration of patient urine samples was characterized using an ISE–based analytical equipment (ADVIA 2400, SIEMENS Healthineers, Erlangen, Germany) at Keimyung University Dongsan Hospital. We used two measurement methods and urine samples from four patients to evaluate the detection of Na^+^ ions using G–ISFET–ISM with Ag/AgCl–RE and FG–RE.

**Figure 6:**
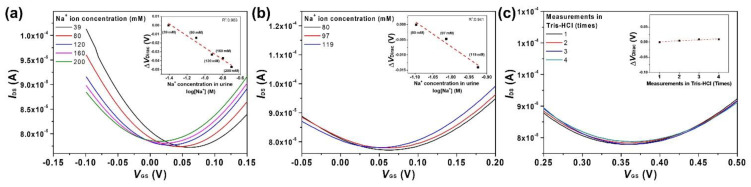
Detection of Na^+^ ions in real human patient urine samples using G–ISFET–ISM with FG–RE. Linear fits were used to extract sensitivity: (**a**) *I*_DS_–*V*_GS_ plots at different Na^+^ concentrations in the same urine sample (added by titration); (**b**) *I*_DS_–*V*_GS_ plots of three different urine samples; and (**c**) *I*_DS_–*V*_GS_ of G–ISFET–ISM with FG–RE in Tris–HCl buffer between measurements of three different patient urine samples.

There are several different ions and substances in human urine. These ions and substances interfere with the ability of G–ISFET–ISM with FG–RE to detect Na^+^ ions. Moreover, the concentrations of such ions and substances differ per urine sample. For the first measurement method, we used the same urine sample and only changed the Na^+^ ions concentration in the urine. The concentration of Na^+^ ions in a unit of urine was 39 mM (S039), which was increased to 200 mM by titration in 40 mM steps. The Na^+^/K^+^ ratio was initially 1.84 and was increased to 9.43. Figure 6a shows the results depending on the Na^+^ ion concentration upon dissolving NaCl in the urine sample. *V*_Dirac_ shifted towards more negative values as the concentration of Na^+^ ions in the urine sample increased. G–ISFET–ISM with FG–RE exhibited a sensitivity of −0.29 mV/mM for Na^+^ ions in the urine sample. The comparative sensitivity to Na^+^ ions of G–ISFET–ISM with Ag/AgCl–RE is shown in Supplementary Figure S7a.

For the second measurement method, we independently tested urine samples from different patients (S037, S047, and S054). The concentration of Na^+^ ions in each urine sample was 97, 80, and 119 mM, respectively. Each urine sample contained a different ionic composition (K^+^, Cr^3+^, and Cl^−^). In the presence of interfering ions and substances in urine, G–ISFET–ISM with FG–RE could detect Na^+^ ions with a sensitivity of −0.36 mV/mM (Figure 6b). The sensitivity of G–ISFET–ISM to Na^+^ ions was higher with the second method (detecting Na^+^ ions in different patient samples) than the first method (detecting Na^+^ ions in the same patient sample). The reason for the higher shift in *V*_Dirac_ can be attributed to two phenomena: (1) the reaction of FG–RE with other ions and the adsorption of substances in urine on the FG–RE surface; and (2) the adsorption of substances in urine onto the ISM surface.

To understand the cause for the increase in sensitivity to Na^+^ ions, we compared the *I*_DS_–*V*_GS_ plots of G–ISFET–ISM with FG–RE in the Tris–HCl buffer among the different urine samples. There was no shift of *V*_Dirac_ (four counts) measured in Tris–HCl buffer before and after the measurement of Na^+^ ion in the each patient urine sample, as shown in Figure 6c. Normally, the concentrations of K^+^ and Ca^2+^ ions excreted through urine are 25–40 and 10–15 mM, respectively [30,41]. The pH value of human urine is pH 4.5–7.8 [44]. These ions may interfere with the detection of Na^+^ ions by G–ISFET–ISM with FG–RE, and the concentrations of such interfering ions differ per urine sample. However, G–ISFET–ISM with FG–RE did not exhibit sensitivity to K^+^, Ca^2+^, and H^+^ ions in the solution in which 100 mM NaCl was dissolved (Figure 5). Therefore, G–ISFET–ISM with FG–RE ignored the effects of K^+^, Ca^2+^, and H^+^ ions. Albumin, which is the main protein in blood, can be excreted through urine [45,46]. Albumin can be precipitated and adsorbed on the ISM surface of G–ISFET–ISM or FG–RE. The absorption of albumin on FG–RE affected the detection of electrical signals. However, the graphene surface with fluorine functional groups inhibited the adsorption of albumin [47,48], and thus the albumin in the urine samples were not adsorbed onto the FG–RE surface.

We considered the adsorption of substances on the ISM surface of G–ISFET–ISM because the sensitivity of G–ISFET–ISM with Ag/AgCl–RE to Na^+^ ions also increased with the second method (Supplementary Figure S7b). The other substances excreted through the kidney appeared to have been adsorbed onto the ISM surface of G–ISFET–ISM, which led to the difference in *V*_Dirac_ between urine samples. We think that if the sensor tip is limited to single–use applications, this shift will be minimized for Na^+^ ion detection.

## 4. Conclusions

In order to realize a disposable sensor with a two–dimensional structure for detecting Na^+^ ions, a new reference electrode based on graphene was proposed. The FG–RE could be used as a reference electrode based on improved chemical stability through fluorination with fluorobenzene. A fluorinated graphene electrode proved the possibility of using the reference electrode by comparing the measurement results using Ag/AgCl–RE. G–ISFET–ISM and FG–RE were integrated on a PCB designed as an SD card to fabricate the Na^+^ ion sensor. G–ISFET–ISM with FG–RE was able to selectively detect Na^+^ ions. To confirm the possibility of practical use, we detected Na^+^ ions in real human urine samples. Based on the results, G–ISFET–ISM with FG–RE exhibited high sensitivity, linearity, and selectivity in the detection of Na^+^ ions in human patient urine. A typical 24 h urine analysis method collects urine from a patient for 24 h and stores it in a refrigerator. This method is very uncomfortable for patients. If the conventional 24 h urine analysis method were to be replaced with our sensor, measuring and recording of the Na^+^ ion concentration each time, the patient would be comfortable without the need to collect urine for 24 h and the accuracy of the 24 h urine analysis method will be increased.

## Figures and Tables

**Table 1:** The ion concentrations (Na^+^, K^+^, Cl^−^, and Cr^3+^) and Na^+^/K^+^ ratio in patient urine samples.

Subject No.	u–Na^+^ (mM)	u–K^+^ (mM)	Na^+^/K^+^ Ratio	u–Cl^−^ (mM)	u–Cr^3+^ (mM)
S037	97.0	25.0	3.88	39.0	56.6
S039	39.0	21.2	1.84	39.0	56.6
S047	80.0	29.1	2.75	38.0	105.1
S054	119.0	28.8	4.13	134.0	79.5

**Table 2:** Comparison of recent G–ISFETs to detect specific ions.

Graphene–ISFET Channel	Detecting Ion/Sensing Range	Reference Electrode	Sensitivity	Ref.
Mechanical exfoliation	H^+^/pH 1–10.5	gate–free	30.8 Ω/pH	[31]
	H^+^/pH 4–10	gate–free	2.13 kΩ/pH	[32]
	H^+^/pH 6–9	Ag/AgCl	17 mV/pH	[33]
	H^+^/pH 4–8.2	Ag/AgCl	30 mV/pH	[34]
Mechanical exfoliation (without ISM)	K^+^, Na^+^/0–10^−3^ M	Ag/AgCl	−	[29,35]
Chemical exfoliation (rGO))	H^+^/pH 6–9	Ag/AgCl	29 mV/pH	[36]
Chemical exfoliation (rGO + oxygen plasma)	H^+^/pH 1–13	Ag/AgCl	57 mV/pH	[37]
Epitaxial growth	H^+^/pH 3–12	Ag/AgCl	19.1 mV/pH	[38]
Chemical vapor deposition (CVD) growth	H^+^/pH 1.2–9	Ag wire	22 mV/pH	[39]
CVD growth + oxygenation (plasma)	H^+^/pH 5.3–9.3	HfO_2_	57.5 mV/pH	[40]
	H^+^/pH 4–10	Ag/AgCl	19.4 mV/pH	[22]
	H^+^/pH 4–10	FG–RE (plasma)	18.2 mV/pH	[22]
CVD growth + ISM	K^+^, Na^+^, NH_4_^+^, NO_3_^−^, SO_4_^2−^, HPO_4_^2−^, and Cl^−^/10^−6^–10^−1^ M	Ag/AgCl	Sensitivity depends on ions (Δ*I*_DS_)	[14]
CVD growth + fluorination (fluorobenzene)	H^+^/pH 4–10	Pt wire	<1 mV/pH	[19]

## Data Availability

The data presented in this study are available on request from the corresponding author.

## Supplementary Materials

The following are available online at https://www.mdpi.com/2079-4991/11/3/787/s1, Figure S1. The fabrication of G–ISFET–ISM for detection of Na^+^ ions. Figure S2. The pH sensitivity of (a) PG–ISFET and (b) FG–ISFET with Ag/AgCl–RE in Carmody buffer. Figure S3. (a) *I*_DS_–*V*_DS_ characteristics of G–ISFET–ISM with FG–RE or Ag/AgCl–RE in Tris–HCl buffer solution; (b) *I*_DS_–*V*_DS_ characteristics of G–ISFET–ISM with FG–RE depending on Na^+^ ions concentration. Figure S4. Evaluation of G–ISFET–ISM with Ag/AgCl–RE according to changes in Na^+^ ions concentration: (a) *I*_DS_–*V*_GS_ and (b) real–time detection. Figure S5. Sensitivity of G–ISFET–ISM with Ag/AgCl–RE to (a) K^+^ and Ca^2+^ ions in Tris–HCl buffer, in which 100 mM NaCl was dissolved; (b) sensitivity to pH in Carmody buffer; and (c) sensitivity to Na^+^ ions in Tris–HCl buffer, in which 100 mM KCl was dissolved. Figure S6. *I*_DS_–*V*_GS_ of G–ISFET–ISM with FG–RE (a) glucose; (b) lactate; (c) bicarbonate; and (d) Mg^2+^ ions in Tris–HCl buffer solution; (e) Sensitivity of G–ISFET–ISM with FG–RE to glucose, lactate, bicarbonate, and (f) Mg^2+^ ions. Figure S7. Detection of Na^+^ ions in real human patient urine samples using G–ISFET–ISM with Ag/AgCl–RE: (a) *I*_DS_–*V*_GS_ at different concentrations of Na^+^ in the same urine sample (added by titration); (b) sensitivity to Na^+^ ions in three different urine samples; and (c) ∆*V*_Dirac_ of G–ISFET–ISM measured in Tris–HCl buffer between measurements of three different urine samples.
