# Supplementary material for: Two–Dimensional Disposable Graphene Sensor to Detect Na+ Ions
Source: Nanomaterials (Basel). 2021 Mar 19;11(3):787. doi: 10.3390/nano11030787 (PMC8003527; doi:10.3390/nano11030787)
Supplement: Supplementary file 1 [file nanomaterials-11-00787-s001.pdf]

Supplementary material

Two-Dimensional Disposable Graphene Sensor to Detect Na^+ Ions

Hong Gi Oh [1,†], Dong Cheol Jeon [1,†], Mahmudah Salwa Gianti [1], Hae Shin Cho [1], Da Ae Jo [1], Muhammad Naufal Indriatmoko [1], Byoung Kuk Jang [2], Joon Mook Lim [3], Seungmin Cho [4] and Kwang Soup Song [1,*]

[1] Department of Medical IT Convergence Engineering, Kumoh National Institute of Technology, Gumi 39177, Korea; oh558@naver.com (H.G.O.); vcaptin@kumoh.ac.kr (D.C.J.); algi.salwa@gmail.com (M.S.G.); nunnunnun@naver.com (H.S.C.); jda226@gmail.com (D.A.J.); muhammadnaufal9.1@gmail.com (M.N.I.)
[2] Department of Internal Medicine, Keimyung University School of Medicine, Daegu 41931, Korea; Jangha106@gmail.com
[3] Department of Creative Convergence Engineering, Hanbat National University, Daejeon 34158, Korea; JoonMookLim@gmail.com
[4] MCK Tech Co., Ltd., Daejeon 34013, Korea; seungmin.cho@mcktech.co.kr
* Correspondence: kssong10@kumoh.ac.kr; Tel.: +82-54-478-7435
† These authors contributed equally to this work.

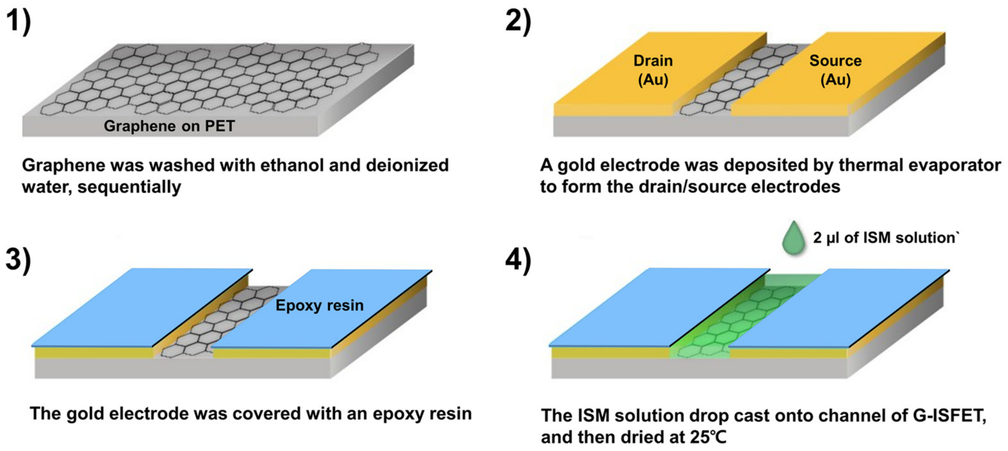

Figure S1. The fabrication of G-ISFET-ISM for detection of Na^+ ions.

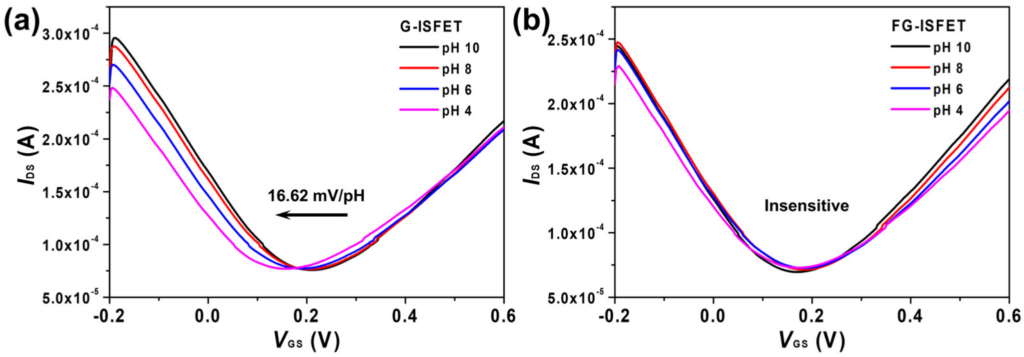

Figure S2. The pH sensitivity of (a) PG-ISFET and (b) FG-ISFET with Ag/AgCl-RE in Carmody buffer.

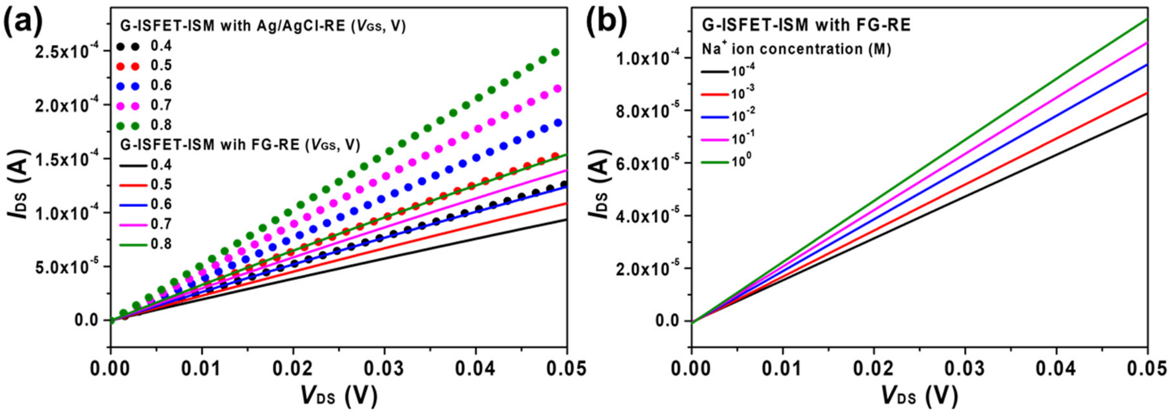

Figure S3. (a) I_{DS}-V_{DS} characteristics of G-ISFET-ISM with FG-RE or Ag/AgCl-RE in Tris-HCl buffer solution; (b) I_{DS}-V_{DS} characteristics of G-ISFET-ISM with FG-RE depending on Na^+ ions concentration.

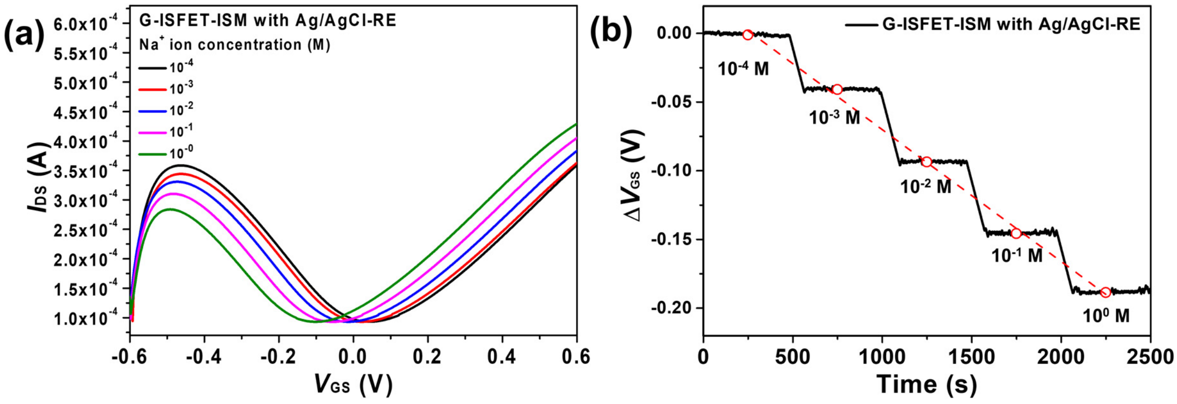

Figure S4. Evaluation of G-ISFET-ISM with Ag/AgCl-RE according to changes in Na^+ ions concentration: (a) I_{DS}-V_{GS} and (b) real-time detection.

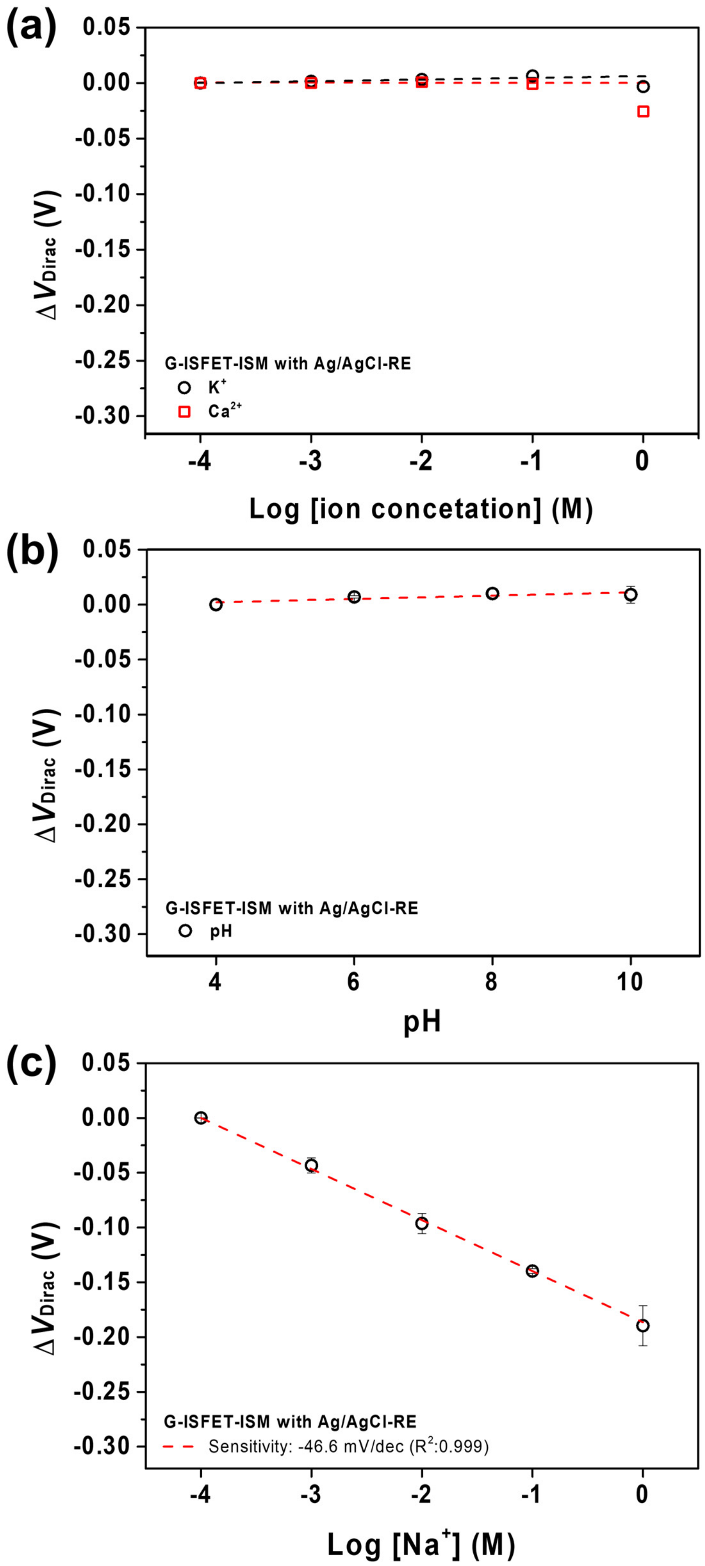

Figure S5. Sensitivity of G-ISFET-ISM with Ag/AgCl-RE to (a) K^+ and Ca^{2+} ions in Tris-HCl buffer, in which 100 mM NaCl was dissolved; b) sensitivity to pH in Carmody buffer; and c) sensitivity to Na^+ ions in Tris-HCl buffer, in which 100 mM KCl was dissolved.

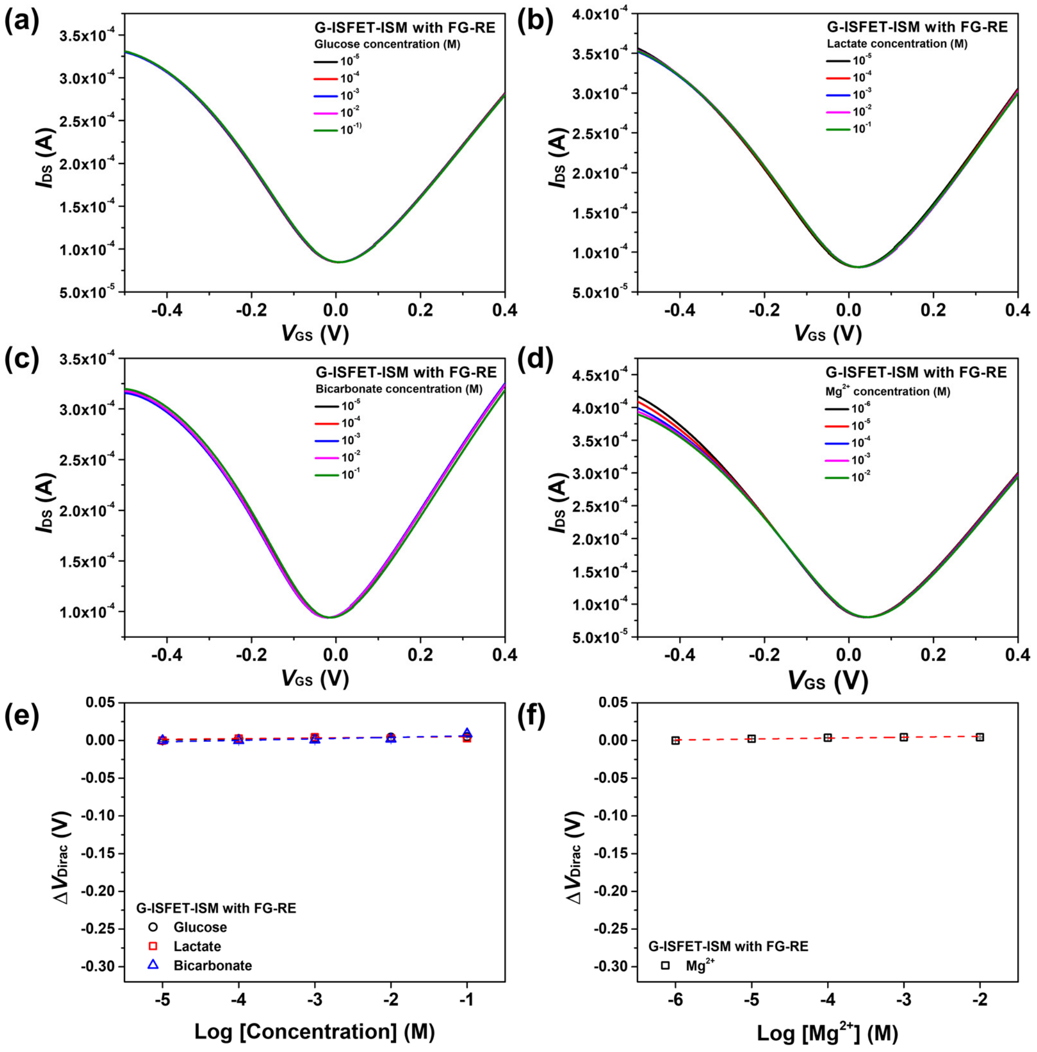

Figure S6. I_{DS}-V_{GS} of G-ISFET-ISM with FG-RE (a) glucose; (b) lactate; (c) bicarbonate;, and (d) Mg^{2+} ions in Tris-HCl buffer solution; (e) Sensitivity of G-ISFET-ISM with FG-RE to glucose, lactate, bicarbonate, and (f) Mg^{2+} ions.

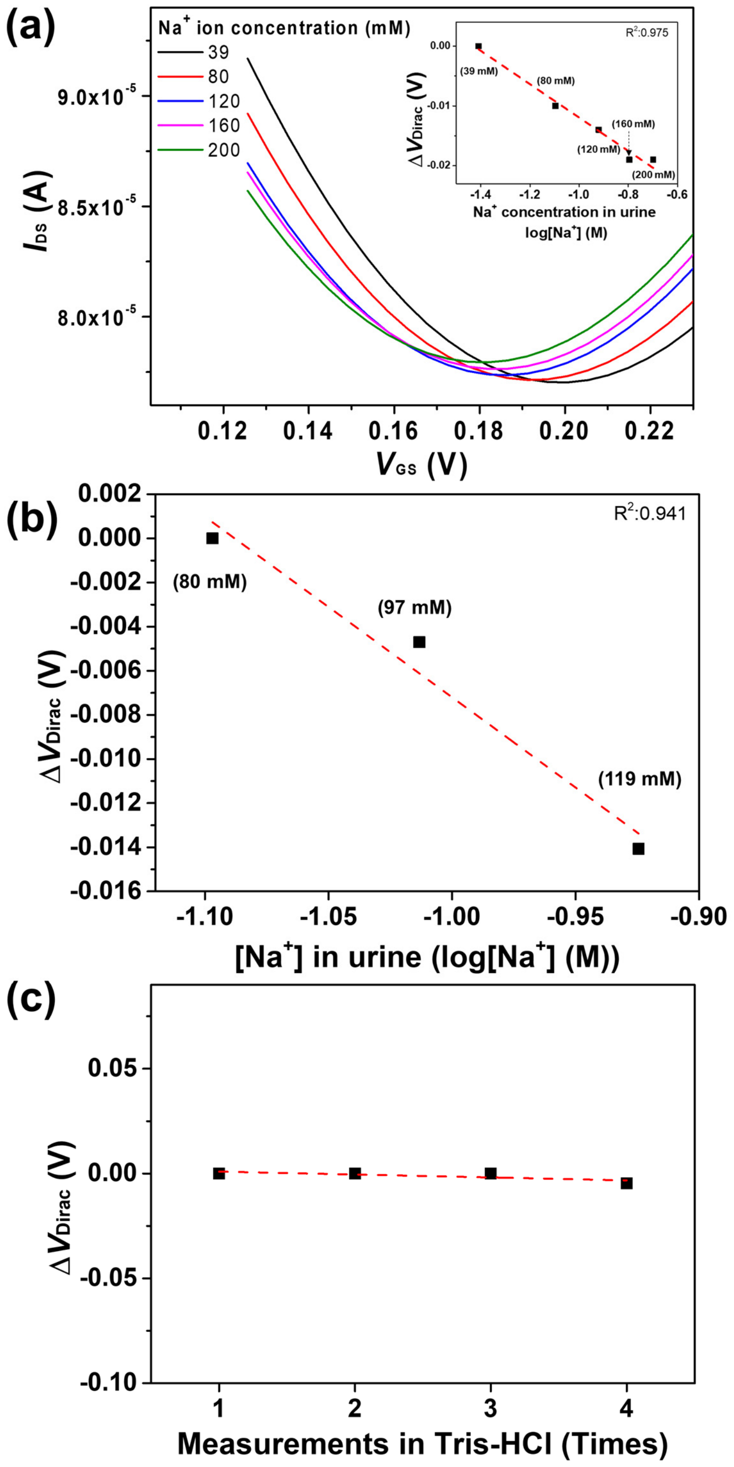

Figure S7. Detection of Na^+ ions in real human patient urine samples using G-ISFET-ISM with Ag/AgCl-RE: (a) I_{DS}-V_{GS} at different concentrations of Na^+ in the same urine sample (added by titration); (b) sensitivity to Na^+ ions in three different urine samples; and (c) ΔV_{Dirac} of G-ISFET-ISM measured in Tris-HCl buffer between measurements of three different urine samples.
